# C-751-08. The Impact of Social Drivers of Health on Social Reintegration After Burn Injury in Children

**DOI:** 10.1093/jbcr/irag033.013

**Published:** 2026-04-06

**Authors:** Sravya Gourishetti, Colleen M Ryan, Robert L Sheridan, Jeffrey C Schneider, Huan Deng, Elizabeth Flores, Kimberly Roaten, Andrew Humbert, Caitlin M Orton, Lewis E Kazis

**Affiliations:** Massachusetts General Brigham/ Shriner's Burn Center, Harvard Medical School, Boston, MA; Massachusetts General Hospital, Harvard Medical School, Boston, MA; Shriners Children's - Boston, Boston, MA; Spaulding Rehabilitation Hospital, Mass General Brigham, Harvard Medical School, Boston, MA; Spaulding Rehabilitation Hospital, Harvard Medical School, Boston, MA; Keck School of Medicine, University of Southern California, Los Angeles, CA; Department of Psychiatry, University of Texas Southwestern Medical Center, Dallas, TX; University of Washington Department of Rehabilitation Medicine, Seattle, WA; Department of Surgery, University of Washington Medicine Regional Burn Center, Harborview Medical Center, Seattle, WA; Boston University School of Public Health, Boston, MA

## Abstract

**Introduction:**

Differences in the risk and causes of burn injury among children have been associated with various social determinants of health (SDOH). Studies indicate that children living in socioeconomically disadvantaged neighborhoods experience higher rates of burn injuries. However, the impact of SDOH on social reintegration of children living with the effects of burn injuries has not been well studied. This study aims to assess differences in social reintegration after burn injury in children based on their pre-injury SDOH.

**Methods:**

Pediatric burn survivors aged 8 to 17 years old participating in a national longitudinal multicenter database between 2015 and 2025 were included in this study. Participants were divided into three insurance groups as reported from their medical record: private (private insurance/HMO/PPO/Pre-paid/Managed Care, self-pay, VA), public (Medicare, Medicaid, Champus/Tri-Care), and other (other, philanthropy, missing/unknown) as a surrogate for SDOH. Self-reported PROMIS Peer Relationship scores assessing quality of their perceived relationships with their friends and acquaintances at 6-, 12-, and 24- months post-injury were analyzed. Reported Peer Relationship scores are converted to T-scores with mean of 50 and standard deviation of 10 and used for analysis. A multivariate linear mixed effects regression model was used to assess the association between a child’s insurance payor and PROMIS Peer Relationship scores, controlling for demographic and injury variables.

**Results:**

This study included 162 children with mean age of 12.6 years old. These children were predominantly male (n = 116), white (n = 68), and incurred fire/flame burns (n = 114). Of which, 41 participants had private insurance, 55 had public insurance, and 65 had other payor status. Children with public insurance had an average PROMIS Peer Relationship score 4.7 points less than (95% CI -9.3 to -0.08) children with private insurance (*p* value = 0.046) across the time periods, indicating worse relationships with their peers. Being male, white, younger, and non-Hispanic trended toward higher peer relationship scores, but without statistical significance. When site of care was added to the regression model, the relationship between Peer Relationship scores and the insurance groups became insignificant.

**Conclusions:**

Child burn survivors with public insurance had more difficulty integrating with their peers after burn injury compared to those with private insurance, but this may be variable based on a child’s site of burn care.

**Applicability of Research to Practice:**

This study contributes to early identification of vulnerable subgroups who may require more support during their recovery.

**Funding for the study:**

This work was supported by Shriners Children’s (Grants #72000 and 79 145) and the National Institute on Disability, Independent Living, and Rehabilitation Research (Grants #90DPBU0001 and 90DPBU0008).

